# Live cell microscopy: From image to insight

**DOI:** 10.1063/5.0082799

**Published:** 2022-04-21

**Authors:** Andreas P. Cuny, Fabian P. Schlottmann, Jennifer C. Ewald, Serge Pelet, Kurt M. Schmoller

**Affiliations:** 1 Department of Biosystems Science and Engineering, ETH Zurich, 4058 Basel, Switzerland; 2 Swiss Institute of Bioinformatics, 4058 Basel, Switzerland; 3 Interfaculty Institute of Cell Biology, University of Tuebingen, 72076 Tuebingen, Germany; 4 Department of Fundamental Microbiology, University of Lausanne, 1015 Lausanne, Switzerland; 5 Institute of Functional Epigenetics, Helmholtz Zentrum München, 85764 Neuherberg, Germany; 6 German Center for Diabetes (DZD), 85764 Neuherberg, Germany

## Abstract

Live-cell microscopy is a powerful tool that can reveal cellular behavior as well as the underlying molecular processes. A key advantage of microscopy is that by visualizing biological processes, it can provide direct insights. Nevertheless, live-cell imaging can be technically challenging and prone to artifacts. For a successful experiment, many careful decisions are required at all steps from hardware selection to downstream image analysis. Facing these questions can be particularly intimidating due to the requirement for expertise in multiple disciplines, ranging from optics, biophysics, and programming to cell biology. In this review, we aim to summarize the key points that need to be considered when setting up and analyzing a live-cell imaging experiment. While we put a particular focus on yeast, many of the concepts discussed are applicable also to other organisms. In addition, we discuss reporting and data sharing strategies that we think are critical to improve reproducibility in the field.

## I. INTRODUCTION

Ever since the days of Antonie van Leeuwenhoek and Robert Hooke, progress in cell biology has been tightly interwoven with technical advances in microscopy approaches. This technical progress is not only limited to the microscope optics but also includes the ever-growing list of available fluorophores and fluorescent proteins, as well as hard- and software for image acquisition and downstream analysis.

The rise of microfluidics to grow cells in a stable environment, together with the explosion of computational power enabling automated cell segmentation and tracking, now allows live-cell imaging over multiple hours or even days. These live-cell imaging setups provide a powerful tool to study dynamic cellular processes on a single cell level. Aside from giving a direct readout of cellular physiology and behavior, fluorescence microscopy can be used to simultaneously obtain insight into molecular processes. Fluorescence microscopy can not only be used to visualize spatiotemporal localization of subcellular structures and proteins but can also provide quantitative insights, for example, on the amount of fluorescent molecules. However, there are many steps on the way to obtaining quantitative live-cell imaging data, including many pitfalls that may introduce artifacts. Unfortunately, consensus for best practice and standardized approaches for live-cell imaging experiments and data analysis are hardly available. This makes quantitative comparison of data obtained by different research groups often difficult and also constitutes a significant barrier for scientists—especially those new to the field—to use the full potential of their data.

Many aspects of live-cell imaging have initially been pioneered using unicellular model organisms such as budding or fission yeast. They are easy to cultivate, grow fast, and their immobility and simple geometry enables automated cell segmentation and long-term tracking. Due to their small diameter of a few micrometers, single wide-field images focused on the center of the cell are often sufficient to capture the relevant information required for a certain biological question, alleviating the need for complex 3D imaging and segmentation. Finally, the powerful genetic tools available make fluorescent tagging of proteins a fast and straightforward process.

**FIG. 1.**
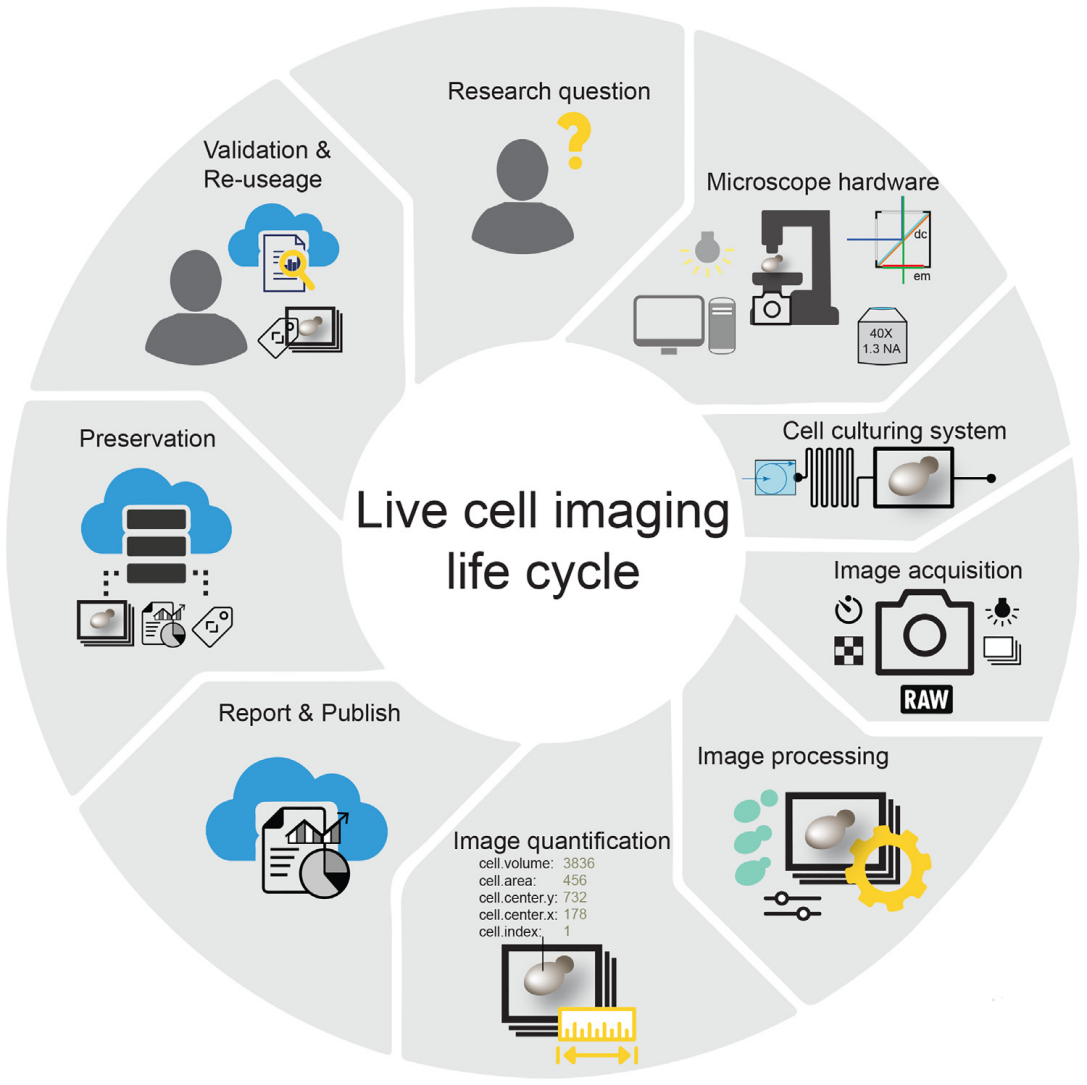
Illustration of the many steps needed to successfully address a biological question using live-cell microscopy and preserve the data for future work.

Here, we review the aspects we consider important to successfully perform and analyze quantitative live-cell-imaging experiments. Following our own expertise, we look at the field with an emphasis on budding yeast, but most of the concepts discussed can be directly transferred to imaging of other organisms. We aim to provide a holistic overview ranging from hardware setups and experimental design to image post-processing, data analysis, and reporting (Fig. 1). We hope that this guide will be useful to experienced microscopists and scientists new to live-cell imaging alike.

## II. MICROSCOPY HARDWARE

**FIG. 2.**
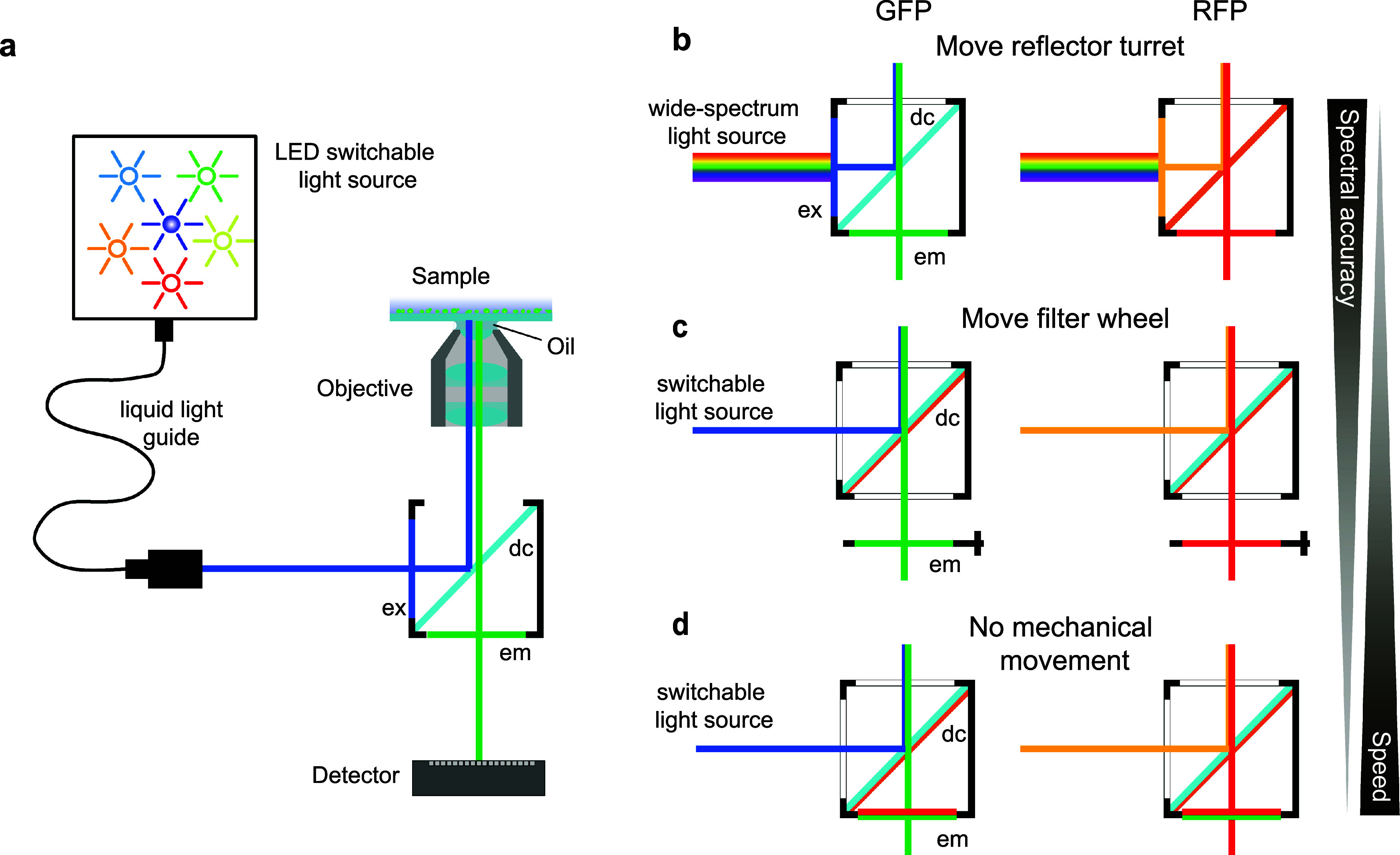
(a) Scheme of an epi-fluorescence microscopy setup. The LED light source is coupled into the microscope using a liquid light guide. The excitation light is filtered by the excitation filter (ex) reflected by the dichroic mirror (dc) and sent into the objective. The fluorescent light generated by the sample is collected by the objective and passes through the dichroic mirror and an emission filter before reaching the detector. (b)–(d). Different filter configurations to detect GFP (right) and RFP (left). In (b), the filters and dichroic are placed in separate filter cubes and the switching of the reflector turret allows one to change the detection channel. In (c) and (d), the excitation light is selected and filtered directly by the LED housing. In (c), a multiband dichroic mirror is used and the excitation filters placed on a separate emission wheel positioned in front of the detector filter the emitted light. In (d), both dichroic and emission filters are multiband optics and the channel selection comes solely from the change of the excitation light.

The development of fluorescent proteins has opened up a whole new area in the microscopy field by allowing to follow in real time the behavior of endogenously tagged proteins. Epi-fluorescence microscopy is often the technique of choice to monitor the behavior of single living cells (Fig. 2). Other imaging modalities, such as scanning or spinning disk confocal microscopy, can offer a better spatial resolution but generally incur more photo-damage to the sample.^1^ In addition, image acquisition by epi-fluorescence microscopy is typically faster by illuminating the whole field of view at once. Technical developments in the recent decades have largely contributed to transforming fluorescence microscopy into a fully quantitative technique. The stability of light sources combined with hardware auto-focusing systems makes it relatively straightforward to perform time-lapse measurements that last multiple hours or even several days.^2–10^ In this section, we will highlight the latest technical development for various components of imaging systems, which should be kept in mind when setting up a new microscope or upgrading older equipment.

### A. Automation

In order to automate the acquisition process, a fully motorized microscope is required. Multiple software from microscope vendors can be used; however, open-source programs supporting the major brands of microscope and accessories and offering simple graphical user interfaces (GUI) complemented by extensive scripting possibilities offer a convincing alternative.^11–14^ The motorized components allow automatic switching of the illumination settings in order to record different fluorescent probes from the same field of view. In addition, a motorized xy stage and a z-drive allow parallel imaging of multiple positions during a single (time-lapse) experiment. If fast z-stack acquisitions are necessary, a piezo-stage should be added to the system.

In quantitative live single cell experiments, an important parameter to consider is the number of cells measured. A larger number of measured cells will allow one to draw stronger conclusions based on an increased statistically significance. The ability to visit multiple positions within the sample is, therefore, crucial.

### B. Light source

LED-based fluorescence excitation sources have become the standard for epi-fluorescence microscopy.^15^ Compared to older mercury or metal halide lamps, they offer numerous advantages: First of all, they have very long lifetimes, and their intensities display limited decrease over time. They also have excellent day-to-day stability. Another key advantage of LED based illumination sources is that they can be switched on and off electronically within tens of microseconds without the need for mechanical shutters. This enables tight synchronization between illumination and image acquisition to limit photobleaching (see Sec. III C). The first LED-based systems offered only a limited number of fluorescent channels. Current state-of-the-art light sources provide typically more than six different colors, which are usually sufficient to excite the full range of fluorophores used in live-cell imaging experiments.

### C. Optical filters

Epi-fluorescence microscopy relies on the use of three different optical filters: the exciter, the beam splitter or dichroic mirror, and the emitter [Fig. 2(a)]. The combination of these three filters allows for specific detection of the fluorophore of interest. For example, BFP, GFP, RFP, and iRFP or CFP, YFP, RFP, and iRFP can be used without much bleed-through between individual fluorophores. Due to the broad spectra of fluorescent proteins, it becomes challenging to combine more than four different probes in the same sample. However, by combining very specific filter sets, a recent study has imaged six fluorescent proteins in the same cell.^2^

LED-based light sources emit in a specific wavelength range. The emitted light still needs to be cleaned up in order to avoid that the tail of the excitation light perturbs the detection of the weaker fluorescent signal. These excitation filters are typically placed in the LED housing rather than in the microscope itself.

Two different combinations of filters can be used: single band or multi-band [Figs. 2(b)–2(d)]. A single band filter set offers a more specific detection of the fluorophore of interest and can provide more signal since the excitation and emission bandpass filters are usually broader [Fig. 2(b)]. However, the drawback of using individual filter sets for each fluorophore is that the imaging of multiple dyes in the sample requires rotation of the filter turret of the microscope. This mechanical change is relatively slow and can have a significant impact on the throughput of an experiment.

By pairing a multi-band dichroic mirror with a multi-band emitter, multiple channels can be recorded by simply changing the active LED [Fig. 2(d)]. This is the fastest measurement strategy because no moving parts are changed. However, some crosstalk between the different channels can occur. A compromise can be achieved by using a multi-band dichroic in combination with single band emission filters placed in a filter wheel [Fig. 2(c)]. The use of single band filters allows a more specific detection of each fluorophore compared to a multi-band emitter. The rotation of the filter wheel slightly slows down the acquisition, but it is faster than the movement of the turret of the microscope.

### D. Objective

In order to maximize the sensitivity of a microscopy setup, it is important to select objectives with a high numerical aperture (NA). The NA represents the angle of collection of the fluorescence light emitted by the sample and determines the ideal spatial resolution of the image

$$D=\frac{\lambda }{2\;NA}.$$

For example, for a wavelength (λ) of 500 nm and an objective with an NA of 1.4, the theoretical spatial resolution (D) corresponds to 178 nm.^16^ Oil immersion objectives with high magnification possess the highest NA and, thus, the best sensitivity and resolution. However, a trade-off between resolution and size of the field of view has to be considered; for example, the field of view imaged with a 100× objective will be more than six times smaller than the one obtained with a 40× objective. In many experiments where spatial accuracy in xy dimensions and also along the z-axis is not critical, it might be advantageous to use a 60× or a 40× objective in order to image a larger portion of the sample and, thus, collect measurements from more single cells. Similarly, lower magnification objectives typically have a larger depth of field,^16^ which can be an advantage for some applications (see Sec. VI), and yield a higher signal-to-noise ratio at a given NA and light dose.^17^

When choosing a suitable objective, wavelength-dependent aberrations have also to be considered: Specifically, wavelength-dependent differences of the focal points along the z-axis lead to *axial chromatic aberration*, and wavelength-dependent differences in the magnification lead to *lateral chromatic aberration.*^16^ Apochromat objectives offer improved performance compared to achromat or single lens-based objectives and can correct the focal point mismatch of axial chromatic aberration. Moreover, image processing algorithms to correct axial and lateral chromatic aberration should be used for fluorescent microscopy studies that require precise positional quantification of multicolor markers such as protein co-localization studies.^18,19^ Additional off-axis aberrations, such as coma, astigmatism, field curvature, and distortion, are typically corrected by the objective manufacturer.

In addition to choosing a well-suited objective, it is important to match the camera to the resolution of the optical system. Using a 100× objective, the spatial resolution of 178 nm will correspond to 17.8 *μ*m in the projected image on the detector. Based on the Nyquist criterion, sampling with at least more than two times the resolution is required.^16,20^ Thus, in our example, the pixel size of the camera should be close to 9 *μ*m. However, oversampling the image with much smaller pixels will not bring additional resolution while it can decrease the sensitivity of the detection.

### E. Camera

A new generation of cameras with sCMOS technology has emerged in the recent decade and has become the reference for epi-fluorescence imaging, replacing the common CCD cameras. The latest models of sCMOS cameras with back-illuminated sensors can overcome 90% quantum efficiency. However, at very low photon counts, EM-CCD cameras still offer better signal-to-noise ratio. One main advantage of the sCMOS chips is that they often have multiple millions of pixels offering up to a four times larger imaging area than traditional CCD cameras. The increased field of view provides the opportunity to image more cells simultaneously. However, in order to accommodate even larger sensors, microscope vendors will have to increase the field of view of the apparatus beyond the typical 22 mm in order to avoid clipping of images or flatness of field issues (see Sec. V).

### F. Advanced microscopy

Various imaging modalities can be implemented on a microscopy setup with the integration of specific hardware elements. For transmission images, polarizers and prisms before the condenser and after the objective can generate differential interference contrast (DIC) images with enhanced contrast.^21^ For phase contrast microscopy, special objectives with an integrated phase plate are required. In addition to the improved contrast, this imaging modality can be adapted to provide quantitative images, allowing to determine cellular dry mass.^22–24^

Numerous imaging modalities have been developed for fluorescence imaging. For instance, based on Förster resonant energy transfer (FRET),^25^ protein–protein interaction or the activity of biosensors can be monitored.^26,27^ To detect subtle changes in fluorescence emission generated by the FRET process, it is advantageous to equip the microscope with an emission filter wheel to acquire the images with specific combinations of filters (for instance, excitation of the GFP and detection in the RFP channel).^28^ For an improved sensitivity in FRET measurements, fluorescence lifetime imaging microscopy can be implemented. However, it requires typically a more complex setup, including a pulsed laser and a time-resolved detection system.^29,30^

Photo-bleaching or photo-conversion techniques such as FRAP (fluorescence recovery after photobleaching) require a bright laser and scanning mirrors to bleach or photo-activate a specific region of the sample.^31,32^ These techniques can reveal the fast exchange of proteins between cellular compartments and aggregates.^33,34^ Laser excitation setups can also be used for TIRF (total internal reflection fluorescence) imaging by directing the laser beam at the side of a high NA objective. Total internal reflection at the glass-medium interface then generates an evanescent wave in the sample, which excites only the molecules in close proximity of the coverslip.^35,36^ Local excitation can also be useful for opto-genetic experiments, where protein–protein interactions or gene expression can be triggered by shining light on the sample.^37,38^ Note that for some opto-genetic systems, the light stimulus can be provided by LEDs placed on the transmission arm of the microscope, but this does not allow for spatial modulation of the activation.^39^

More sophisticated microscopy setups, such as scanning or spinning disk confocals, can be necessary to improve the image resolution relative to standard epi-fluorescence.^40,41^ Alternatively, imaging techniques such as light-sheet or lattice light-sheet microscopy, using an excitation beam perpendicular to the detection axis, can provide high-resolution 3D images of live samples while minimizing photo-damage.^42–45^ In order to break the resolution limit imposed by the diffraction of light, various super-resolution techniques have emerged. Structured illumination microscopy (SIM) or Airyscan confocal systems can provide an improvement of two to threefold in resolution.^46,47^ To further improve spatial resolution and obtain single molecule precision, localization microscopy uses multiple cycles of photoactivation and deactivation to reconstruct the position of individual fluorophores in the sample.^36,48,49^

### G. Stability

Motorization of microscopy setups allows recording of high-dimensional time-lapse movies, imaging multiple xyz positions over multiple time points, and combining several imaging channels, including epifluorescence and transmission images. In order to robustly quantify the evolution of cells as a function of time, the overall stability of the system is essential. The repeatability of the xy stage has to be highly precise. In addition, autofocusing systems provide considerable help in maintaining the sample in the focal plane of the microscope. A hardware autofocus measures the position of the sample using the reflection of infrared light at the glass–air (air objectives) or glass–water (oil objectives) interface. This technology is fast and accurate and is the recommended option for imaging of microbial cells, which requires a sub-micron precision in focal plane determination. Alternatively, image-based autofocus can be used, which requires the acquisition of a Z-stack of images to identify the optimal focal plane. Image-based focusing avoids the need for additional hardware and potentially captures additional out-of-focus information. However, this process slows down the acquisition considerably, is not always reliable, and can incur photo-damage to the sample.

An environmental control chamber can also contribute to the overall stability of the microscope setup by minimizing temperature changes that can lead to focus drifts. In addition, the chamber provides a control of the temperature, humidity, and CO_2_ if necessary, which allows maintaining the cells in optimal growth conditions.

In order to obtain reproducible results from day to day, the microscope setup requires proper calibration. Ideally, this routine verification is performed before every experiment. The calibration will ensure the appropriate and homogeneous illumination of the field of view for both transmission and fluorescence modalities. For transmission images, the Koehler alignment of the condenser matters most. For fluorescence illumination, the light is typically brought by a liquid light guide into the system, making the calibration more demanding but providing more stability over time. Using standard fluorescent calibration slides, the light guide can be precisely aligned, and the field flatness can be measured by taking a picture of a uniformly fluorescent sample.

• Maximizing the number of single-cell measurements can be achieved by using a lower magnification objective and a camera with a large sensor.
• A tradeoff between the number of fields of view acquired and the temporal resolution of the experiment has to be found. An optimized fluorescence filter configuration can decrease the amount of time spent at each xy position.
• The stability of the experiment during multi-hour time-lapse experiments can be maintained using an environmental control chamber and autofocusing systems.

## III. SETTING UP THE EXPERIMENT

### A. Cultivating cells under the microscope

In addition to the microscope hardware, live-cell imaging requires means to maintain cells in a controlled environment suited for cell growth. Short-term imaging of live yeast cells can be achieved by simply transferring liquid culture on a microscopy slide, which can be coated to immobilize the cells.^50^ Long-term imaging, however, requires continuous supply of growth media and a method to keep cells in a fixed position. One cheap and easily accessible option is to place diluted yeast cells between microscopy coverslips and agar patches of solid yeast media.^51^ This approach mimics growth on typical agar plates and enables quasi-2D growth of yeast colonies for several hours. However, after a few hours, drying of the agar typically causes drifts, which makes it hard to maintain cells centered and in focus, and expansion of the colony into the third dimension can cause additional problems.

Microfluidics cultivation devices,^52^ either custom built or commercially available, circumvent these problems. Cells are typically trapped in quasi-2D channels between high quality glass and a flexible gas-permeable polymer such as polydimethylsiloxane (PDMS) and provided with a constant flow of fresh media. This way, cells can be maintained in focus and in a steady-state growth condition for many hours and multiple generations.^53^ In addition to enabling longer imaging in steady state conditions, microfluidic devices can also be used to change media conditions during the experiment in a precise manner by using several controllable media inlets, for example, to monitor the dynamics of activation of signal transduction cascades.^54–56^ Controlled mixing of media upstream of the cell chamber allows for even more complicated protocols, such as dynamic concentration ramps or spatial gradients.^57–60^

A typical experiment would be started with single isolated cells spread across the microfluidic chamber, and the experiment would come to a natural end once the chamber is filled with cells. This limits the observable time-frame to not much more than ten divisions. However, using dedicated devices that selectively maintain some cells while flushing away others, individual cells can be imaged throughout their entire replicative lifespan.^7,10,61–64^

Aside from such specialized designs for specific questions, other parameters need to be considered when choosing the correct microfluidics setup: The height of the chamber needs to be adjusted depending on the cell diameter (e.g., different designs for haploid or diploid cells), simultaneous imaging of multiple strains in one experiment can be achieved through parallel chambers, and the ideal flow rate might vary depending on the experiment. For example, low flow rates might be beneficial if a costly reagent needs to be supplemented to the media. On the other hand, higher flow rates might be better suited for certain small molecules, which are otherwise lost through diffusion into the PDMS of the microfluidics device.^65^

After live-cell time-lapse experiments have been performed, an important quality control is to verify that conditions in the chamber and imaging settings do not interfere with cell growth. As will also be discussed in the context of phototoxicity, this can be readily assessed by estimating population doubling times from growing colonies and comparison to that of liquid cultures growing exponentially in the same media. Strong growth rate variability between individual colonies may be an indicator for heterogeneous media flow.

### B. Image acquisition settings

When the cultivation system is ready, the image acquisition needs to be set up. Ideally, image acquisition parameters should remain valid for a whole set of related experiments (for instance, including mutants that might display weaker or higher signals). Thus, small pilot studies to determine the optimal choice of imaging acquisition parameters are often a sensible time investment to avoid downstream problems. Considerations regarding the choice of objectives and type of filters have been described in Secs. II C and II D above. Another important consideration is the trade-off between imaging frequency and the number of positions recorded. The number of positions recorded in one sample and/or the number of samples that can be imaged in parallel will strongly depend on the dynamics of the biological process monitored. Very rapid processes can only be measured in a single field of view, while slower acquisition at the 3–15 min time scale allows recording tens of positions, thereby increasing the number of cells observed and the statistical power of the experiment. A physical limitation to the amount of positions imaged in parallel can arise due to the immersion oil, which may not follow large displacements of the sample, especially at fast stage speed. For very slow biological processes, the automated tracking of the cells in the downstream image processing may set an upper limit to the imaging interval.

The optimal exposure settings (brightness of LED and duration of exposure) must balance (at a minimum) the following four points: First, image saturation must be avoided. Fortunately, this has become easier with recent cameras that offer 16 bits of resolution. Second, the exposure time should be optimized to increase the signal-to-noise ratio. At short exposure time, the fluorescent signal may hardly overcome the endogenous autofluorescence of the sample. Third, the combination of exposure time, imaging frequency, and duration should minimize photobleaching.^66^ If the imaging frequency is too high or the exposure time too long, the decrease in the fluorescence signal might be dominant, so that a quantitative assessment of the process is no longer possible, especially toward the end of the acquisition. Fourth, phototoxicity should be avoided to guarantee the validity of the biological observation.

### C. Phototoxicity

The terms phototoxicity or photomorbidity refer to light-induced damage of the cell. While imaging is, in principle, a “non-invasive” technique, exposing cells to light can be harmful. Phototoxicity is induced by several mechanisms, which are dependent on the wavelength of the light exposure. High-energy (i.e., low wavelength) light below ∼340 nm can directly break chemical bonds, especially those in pyrimidines of DNA, and thereby lead to DNA lesions and mutations. Longer wavelength light can lead to the generation of reactive oxygen species (ROS) and heat dissipation, which in turn damages proteins and membranes.^67–69^ Phototoxicity is mostly caused by the excitation light directly acting on cellular components, independently of a heterologous fluorophore such as GFP. However, fluorescent proteins can also contribute to ROS formation when excited electrons react with dissolved oxygen instead of emitting photons. This usually goes hand in hand with photobleaching. Some fluorophores are more prone to producing and releasing ROS, the most extreme case being KillerRed.^70^

Cells have extensive mechanisms for detoxifying ROS and clearing other photo-induced damage, but as the repair system approaches saturation, cell function becomes affected. One key objective for live-cell imaging experiments is, therefore, to keep light-induced damage to a minimum. Importantly, the tolerance to light is dependent on the nutrient supply, and environmental stresses can amplify phototoxic stress.^17^ Severe phototoxicity is evident if cells stop growing and dividing or change their morphology. Yet subtle changes in physiology will occur already at lower light doses and in shorter times.^17,71^ Therefore, for quantitative analysis of cellular processes, sensitive read-outs to exclude photomorbitity under all assay conditions are needed. Carefully monitoring the growth rates of many cells or colonies is a sensitive readout to determine the maximal light tolerated without obvious physiological effect.^17^ Comparing cells exposed to excitation for fluorescence channels with those only imaged by brightfield or growing in liquid culture allows quantifying phototoxic effects within one experiment. Another important control is comparing experiments with different time intervals between imaging. This can reveal quantitative differences in physiology due to phototoxic stress and will also show if any drifts in the signal are caused by photobleaching.

**FIG. 3.**
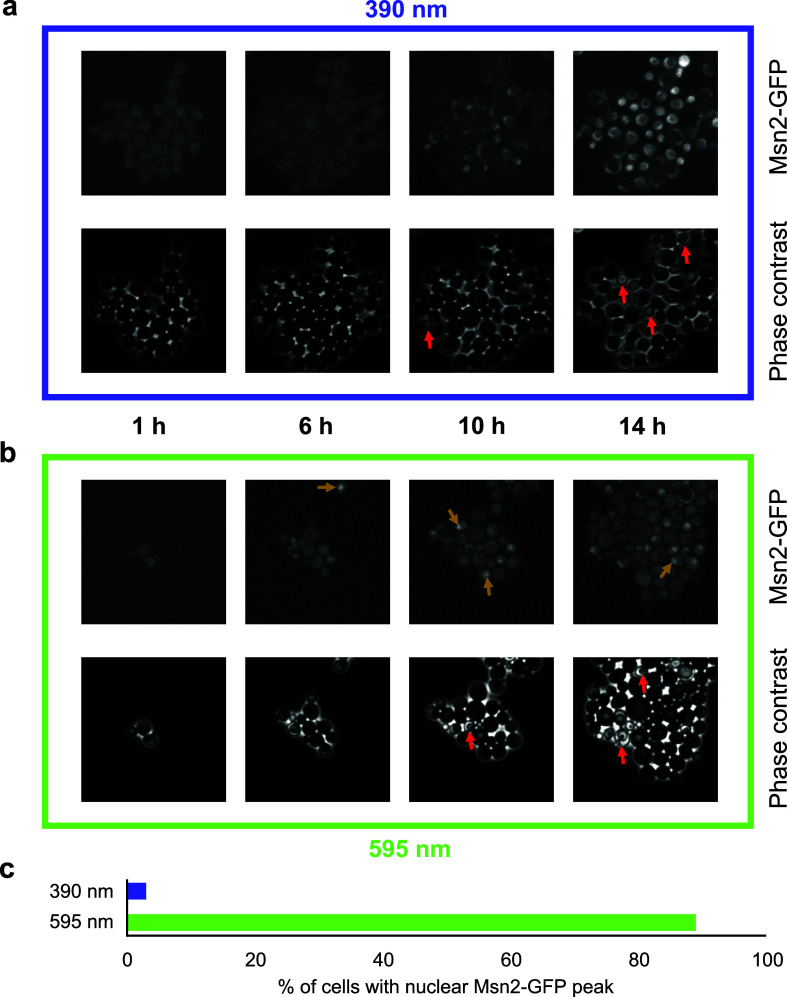
Response to phototoxicity is wavelength dependent. A strain expressing Msn2-GFP was grown in a microfluidic plate with constant flow of the glucose minimal medium. Cells were photo-stressed at levels that induce cell death after ∼10 h and imaged every 5 min. Upper lanes show the GFP fluorescent image, and each lower lane corresponds to phase contrast images. (a) Cells show no Msn2-GFP signal under blue (390 nm) light stress but cell death/stress-induced autofluorescence occurs after ∼10 h. Red arrows: cell death. (b) Cells stressed with yellow (575 nm) light show nuclear Msn2-GFP signal (examples indicated with orange arrows). The signal oscillates; thus, only a fraction of cells shows signal at a single time-point. (c) Most cells stressed with yellow light show nuclear Msn2-GFP at least once over the time course, while cells stressed with blue light do not.

Another method to monitor phototoxicity is using stress responsive proteins that change their amount or localization. To quantify light stress in the green range, Msn2 has been successfully used as a reporter in budding yeast.^72^ However, Msn2 does not respond to blue light (Fig. 3) even at light doses where growth completely stops. Reporters such as fluorescently labeled proteins, such as Rad53 or Yap1, can be monitored to detect DNA damage or to monitor oxidative stress caused by excessive ROS.^73,74^

If phototoxicity is detected, how can it be avoided? Longer wavelength light is less damaging than shorter wavelengths, even when the same total energy is applied, since individual photons can transfer less energy to the biomolecules they collide with. The choice of fluorophores is, therefore, particularly relevant when imaging low-abundant proteins that require more excitation light (see FPbase for a comprehensive list of available fluorophores^75^). For a given wavelength, the key parameter that determines phototoxicity is the total energy applied per exposure,^17^ i.e., the integral of light intensity over exposure time. In practice, however, many instruments do not control the exposure time precisely, such that a delay in turning off the light source often leads to longer light exposure than intended by the experimentalist.^66,76^ As a consequence, choosing lower light intensity at longer set exposure time often causes less damage.^66^ To overcome these delays in hardware control, commercial systems are now offered with TTLs (transistor–transistor logic) controlling the hardware. These TTL-based controls are more precise in setting the “on-time” of the lamp/shutter to exactly the defined exposure time. Also other hardware components can make a major contribution to reducing phototoxicity. For example, a higher sensitivity camera and optimal emission filters allow shortening exposure times. To compare the actual light exposure that cells experience with different hardware configurations and settings, a microscope slide power sensor and other specialized hardware can be used.^17,77^

• Microfluidic devices enable long-term imaging over multiple generations by providing steady media supply and keeping cells in focus. Ideal choice of device depends on experimental needs.
• Microfluidics enable precise media switches and complex stimulation patterns.
• The timing of image acquisition needs to match the dynamics of the biology.
• Choice of fluorophore, hardware, and exposure settings is a balance between signal-to-noise and dynamic range on the one hand, and photobleaching and phototoxicity, on the other hand.
• Environmental stress and nutrient supply can amplify photodamage; therefore, phototoxicity needs to be excluded for every fluorescent protein construct under every assay condition.
• Phototoxicity can affect physiology long before cells stop growing. Growth measurements should be performed as quality control.

## IV. IMAGE PROCESSING

After an experiment has been successfully performed, the obtained raw imaging data have to be analyzed. The goal of the image analysis process is to transform the microscopy images into quantitative single cell measurements. In order to fulfill this task, three consecutive steps have to take place. First, the images must be segmented to identify individual cells. Then the features of the cells are quantified. Finally, the identified cells (or features) are tracked from one frame to the next.

### A. Image segmentation

During segmentation, the microscopy image is converted into a mask, which indicates for each pixel if it belongs to the background or if it is inside a cell. In addition, for each cellular pixel, segmentation should also determine to which cell it belongs. While the human eye is highly trained to recognize various objects, this task is not trivial to automate.^78^ The source image(s) for the recognition of the cells will depend on the sample and the details needed. The analysis of fluorescent images, which have a dark background and where objects display a strong signal, is relatively straightforward. However, because the number of fluorescent channels available in a microscope is limited, segmentation based on transmission images, while more challenging, is often preferred. Nonetheless, additional fluorescent images can be combined with transmission images to identify subcellular structures that cannot be recognized in the transmission image.

In recent years, a number of image analysis pipelines have been developed and are available to segment images (Table I). If these available tools cannot fulfill the more specific requirements of an experiment, then ImageJ, Python, or Matlab have been traditionally used to develop tailored image analysis solutions thanks to the large number of predefined functions for computer vision available.

**TABLE I.** List of image analysis pipelines.

Name	Segmentation method	Source image	Cell type	Specificity	Reference
CellProfiler	Thresholding, edge detection	BF or FL	MC	General segmentation and image quantification platform	97
Cell-ID	Thresholding	BF	BY	Budding yeast quantification and tracking program written in C	91
YeastQuant	Thresholding	BF or FL	BY	Budding yeast quantification and tracking platform combining a FileMaker database for documentation and Matlab program	92
PhyloCell	Thresholding and watershed	BF	BY	Matlab GUI for visualization of the segmentation and phylogeny	94
PombeX	Contour	BF and FL	FY	Combines fluorescent image of nuclei and a brightfield image to segment fission yeast	95
CellX	Graph cuts	BF or FL	MC, BY, FY	Segmentation platform based on border detection applicable to a wide range of specimen	88
CellStar	Active contour	BF	BY	Segmentation and tracking of budding yeast	86
Pomegranate	Thresholding	BF and FL	FY	2D segmentation of fission yeast and 3D reconstruction	96
DISCO	Support vector machine	BF	BY	Segment yeast cells in microfluidic traps	189
Conv-nets	Neural network	PH or FL	MC, Bact	Segmentation of phase images for bacteria and mammalian cells	108
Super-segger	Threshold, watershed and neural network	PH	Bact	Segmentation of rod-shaped bacteria	109
StarDist	Neural network	FL	MC	Identification of Star polygons in images	110
Zcells	Support vector machine	BF	Ec, BY, MC	Segmentation based on a stack of BF images	114
CellBow	Neural network	BF or FL	BY, FY, MC	Network trained on in and out of focus images to detect cells in different layers	190
YeaZ	Neural network	PH or BF	BY	Segmentation and tracking of budding yeast including shape mutants	113
Cellpose	Neural network	BF or FL	MC, BY, FY, Ec	Generalist segmentation platform trained on a diverse array of images	111
Cell-ACDC	Neural network	BF or FL	BY, (MC, FY, Ec)	Framework with GUI for image analysis including segmentation (based on YeaZ or cellpose), tracking, pedigree and cell cycle annotations. Cell cycle annotations optimized for BY, segmentation and tracking compatible with other organisms	120
Trac^X^	⋯	BF, PH or FL	BY, FY, MC, Bact	Generalist tracking software compatible with any segmentation algorithm that provides a segmentation mask; image modality and cell type independent with automated lineage reconstruction. Supports work-flows, scripting and provides a GUI for manual error correction (segmentation, tracking and lineage).	118

Note: Image source: BF: Brightfield; PH: Phase contrast; FL: Fluorescence. Cell type: MC: Mammalian cells; BY: Budding yeast; FY: Fission yeast; Bact: Bacteria; Ec: *E.coli*.

### B. Algorithmic segmentation

A large fraction of image analysis methods are based on a specific algorithm or a combination of them.^78,79^ For instance, to detect bright well-separated fluorescent nuclei over a dark background, a simple thresholding method can be used. Depending on the contrast of the image, the identification of the best threshold can be challenging.^80^ If the fluorescent objects are in close contact, watershed algorithms can prove useful to separate merged segmentation masks, although they tend to generate over-segmented objects.^79,81^ For transmission images, edge detection methods can be used to identify sharp changes in intensity indicating the cell periphery.^82,83^ This detection unfortunately does not result in continuous borders for objects. Thus, more complex algorithms have been implemented using active contours, which grow or shrink an object such that the contour deforms according to the intensity in the image,^84–86^ or graph cuts, which use the intensity profile from the interior to the exterior of the object to define its borders.^87,88^ Active contours or graph cut algorithms typically require an initial guess or seed for each object. These seeds can be obtained by a first segmentation performed by intensity thresholding or edge detection.

In all cases, a set of pre-processing and post-processing steps are required. During pre-processing, the source image can be filtered to remove noise and other aberrations in the data. The post-processing will, for instance, make use of watershedding algorithms^81,89^ and morphological operations to smooth the shapes of the identified objects.^90^

Due to their homogenous shapes, it is relatively straightforward to generate an algorithmic pipeline dedicated to the segmentation of budding^86,91–94^ or fission yeast cells.^95,96^ However, using traditional approaches, few analysis pipelines offer enough flexibility to achieve accurate segmentation of images from a wide diversity of cell types.^88,97^

### C. Machine learning

The development of machine learning has provided new opportunities to analyze microscopy images.^98–101^ Diverse tasks, such as automated classification of phenotypes or cellular structures,^102–104^ artificial labeling,^105^ or image restoration and enhancement,^106,107^ have profited from these advances. Neural networks have also been successfully applied to segmentation tasks.^108–111^ An important part of the work consists in training the network with a set of images, where cells have been precisely segmented. The quality of the ground truth data used for training will ultimately determine the precision of the segmentation.

In a recent successful application of deep learning to the segmentation of budding yeast cells imaged with phase contrast, more than 8000 manually segmented cells were used to train a U-Net-based^112^ convolutional neural network.^113^ This dataset also included images from various mutant strains displaying aberrant morphologies to provide a larger diversity of cell shapes and sizes. Another approach that partially by-passes the tedious annotation of single cells has been devised by the Hersen lab.^114^ A z-stack of bright-field images is acquired, and the change in intensity along the z-axis is used as a signature to define various regions of the sample (background, cell border, cell interior, etc.). Because each image contains millions of pixels, it is sufficient to train the machine learning algorithm on one image with roughly 2000 pixels of each category to obtain an efficient classification of cell types. This modular approach has been successfully used to identify various cell types.

### D. Feature measurements

Once the cells are segmented, numerous features can be quantified for each cell. This includes geometrical features such as the area, the small or long axes, eccentricity or solidity, as well as intensity features in each imaging channel (see also Sec. VI).^97,115,116^ Beyond these basic measurements, a wide range of statistical and texture measurements can be performed and used to characterize an object.^100^ Machine learning with support vector machines or random forest can be used to determine the cell type, the cell-cycle stage, or the developmental status based on the various features obtained to classify cells in different categories.^103^

### E. Tracking

Once every frame of a time-lapse move has been segmented and objects quantified, the tracking process has to connect each cell from one frame to the next. When working with yeast, this process is simplified by the fact that yeast cells are non-motile. Therefore, a simple assignment based on the centroid of each cell or overlapping cell areas between frames can be sufficient.^86,117^ This selection can be further refined by also using information about cellular characteristics such as cell size or fluorescence intensity that should not vary sharply from one time point to the next. These additional parameters can decrease the error when connecting cells in close proximity.^118^

The challenge in the tracking of yeast cells comes from their fast division time. Tracking the lineage of cells can become very complicated after only a few divisions. In order to facilitate the tracking and focus on the cells that are important to monitor, it can be advantageous to perform the tracking backward in time and initiate the algorithm with the last frame of the time-lapse.^93^ Microfluidic chambers can also help to restrict division to the focal plane of the microscope.^53,93^ An alternative strategy to simplify drastically the tracking over long time-lapse is to trap the mother cells in a microfluidic chamber and to wash away the daughter cells^7,119^ (see also Sec. III A). For many applications, manual verification of segmentation and tracking data is still needed. Dedicated software with intuitive GUI and automated error propagation can support this process.^62,120^

• Many platforms are available for the segmentation of yeast cells (Table I). Their performances have been compared by the Hersen lab.^86^ It can be helpful to evaluate their robustness for a particular dataset to find the optimal approach.
• Investing the time in training a neural network for image segmentation can result in robust cell segmentation even in complex samples. Starting from an already pre-trained network can greatly decrease the time required for this step.
• Tracking is relatively straightforward with non-motile cells, but lineage tracing is challenging due to the small size of buds and crowded colonies.

## V. IMAGE CORRECTION

Once cells have been segmented and the required pedigree and cell cycle information have been obtained, the cellular fluorescent signal can be quantified. A key requirement for quantitative analysis is that the recorded intensities of the signal of interest are within the linear regime of the detection. Since intensities can vary between conditions and due to cell-to-cell variability, it is often advisable to anticipate higher signal intensities when initially choosing the experimental settings to avoid saturation. An additional element to consider is how uniform the illumination of the sample is. If there is a noticeable difference between the center and the corner of the images, an image (flatness of field) correction step should be applied. An important first step of quantification is then to separate from the total signal measured the part that can be attributed to sources other than the signal to be quantified. This “nonspecific signal” can be broadly classified into two categories. The first category, which we will refer to as “background fluorescence,” includes all signal not related to the presence of the cell. Besides electronic noise, such background can be caused by fluorescence associated with the media or microfluidics device, as well as additional light sources in the microscopy room. The second category includes all cellular fluorescence occurring independent of the fluorophore of interest, such as autofluorescence, as well as in the case of multi-channel imaging, bleed-through from other fluorophores.

### A. Flatness of field

Ideally, all pixels in the image should receive the same excitation light and be detected with the same efficiency. However, due to limitations of the optical system, objects in the center typically appear brighter than those in the periphery of the image. This uneven illumination called vignetting or shading can, in part, be caused by poor alignment of the optical components and can, thus, be improved by centering the excitation light or the detector.^121^ However, often the vignetting effect is caused by the intrinsic properties of optical components and the larger the field of view, the more important the problem will become. Therefore, this shading will be more apparent when using large sCMOS sensors.

Uneven illumination and detection cause obvious problems for absolute quantification from fluorescence images and can also impair object segmentation. However, correction of this artifact is relatively straightforward. One method consists in measuring a reference image from a uniformly fluorescent sample.^21,122^ Alternative approaches extract the shape of the field flatness from one or multiple sample images.^121,123–125^ In both cases, the sample images can then be divided by the normalized reference image to obtain flattened images, which can be properly segmented and quantified.

### B. Background fluorescence

Since background fluorescence may vary between positions and over time, e.g., due to inhomogeneities in the microfluidic setup, photobleaching of the background, or changes in the external light sources, a local and dynamic subtraction of the background is advantageous. In many cases, a good approach is to use the cell segmentation masks to define a “non-cell” region for any given frame and then subtract from cellular pixel intensities the median signal in this non-cell region to obtain a background-corrected image.

### C. Autofluorescence

While background correction can be achieved without additional control experiments, appropriate correction for cellular autofluorescence requires more effort. However, because autofluorescence can vary strongly between media conditions,^126^ strain background,^127^ and even from cell to cell, it often limits the quantitative resolution of the experiment, and therefore, its contribution has to be carefully taken into account. One important first step is the choice of fluorophore and filters to maximize the signal-to-noise ratio by minimizing autofluorescence.^17^ The website FPbase is a great resource to compare the various properties of different fluorescent proteins.^75^ Typically, in constant environments, budding yeast autofluorescence is low and rather stable in the spectral range of yellow fluorescent proteins,^128^ which makes, for example, eYFP, mCitrine, or mVenus good choices for the endogenous tagging of proteins.

Despite an optimal choice of fluorophores, autofluorescence can be a significant contribution to the overall signal, especially when studying weaker expressed proteins. To correct for the average autofluorescence, control experiments with strains that do not carry the fluorescent protein of interest should then be performed.^129,130^ If the control and fluorescent strains can easily be distinguished, additional comparability can be achieved by measuring a mixed cell population simultaneously. Control cells can then be segmented and tracked, and after background correction, a mean pixel intensity can be calculated and subtracted from the signal of the fluorescent cells.

In addition to a correction of the fluorescent signal by the average autofluorescence, the control experiments—along with downstream analysis—can provide important insight into the cell-to-cell variability of autofluorescence, as well as potential biases due to changes of autofluorescence with for example cell cycle state, cell size, or genetic and environmental perturbations.^8,126^ If careful examination of the signal strength and autofluorescence reveals it as necessary, more complex analysis of the autofluorescence control experiment can be performed to correct for such effects. For example, the cells in the control experiment could be binned according to their size to then calculate the size-dependent autofluorescence intensity.^8^

Finally, to estimate the error due to experiment-to-experiment variability and to assess whether autofluorescence and background subtraction were successful in light of a given type of analysis, it is advisable to perform one (or multiple) additional control experiment with non-fluorescent strains that can be mock-analyzed similar to the strain of interest.

### D. Multicolor imaging

For multichannel imaging of several different fluorophores, an approach analogous to that described for autofluorescence correction can be used to account for potential bleed-through from the other channels (despite optimal filter choice). Here, in addition to a non-fluorescent control, control experiments using strains with single fluorophores or strains that each lack one of the multiple fluorophores can be imaged and quantified to determine the contribution of the other fluorescent proteins to the signal in the channel of interest.^2,131^ This can be absolutely critical in situations where the strength of the signal in one channel is much higher than that in other channels, e.g., due to higher protein expression. In this case, even “weak” bleed-through from the brightest channel can lead to a strong contribution to the overall signal in the other channels.^132^ Inspiration for rigorous correction of bleed-through can also be drawn from the field of flow cytometry, where such compensation for multicolor measurements has been standardized two decades ago.^133^

### E. Deconvolution

For applications that require precise measurements of spatial positions or object volumes, in particular, 3D xyz imaging,^134,135^ deconvolution can be used to reconstruct the true point-like source of the fluorescent signal based on either a theoretical or measured point spread function.^136–138^ The point spread function of a given optical setup can be measured by using fluorescent beads.^137^ However, deconvolution algorithms are computationally expensive and can introduce reconstruction artifacts.^139^ Commercial and open-source deconvolution algorithms have been developed and reviewed in detail.^136,139–141^

• To improve quantification accuracy, field flatness should be rectified.
• Background signal can be corrected by subtracting signal in non-cell areas.
• Autofluorescence can be a major contributor to the signal and change with cell size, cell cycle, and environment. Non-fluorescent control strains can be used to correct for autofluorescence.
• Multicolor measurements can require careful compensation measurements.

## VI. QUANTIFICATION AND INTERPRETATION

### A. Quantifying fluorescent signals

After background and autofluorescence have been corrected for, a meaningful parameter to quantify the cellular signal has to be chosen. In many cases, the biological phenomena studied go along with drastic changes in the signal intensity, and the exact choice of the analyzed parameter does not substantially affect the quantification of this “binary-like” signal. However, for any analysis of smaller, more graded responses, careful consideration has to be given to the metric used to avoid artifacts due to confounding variables such as cell size, cell geometry, or signal localization. In addition, accurate reporting is essential to allow correct interpretation and reproducibility.

One of the most direct readouts of the fluorescent signal is the average pixel intensity (after background and autofluorescence correction). However, as described below, depending on the microscopy setup, the meaning of the pixel intensity can vary, which makes interpretation—especially across studies—difficult. Ideally, the quantification should, therefore, correspond as closely as possible to a meaningful physical variable such as fluorophore amount or concentration.

To obtain absolute measurements of fluorophore amounts, calibration measurements are required that quantify the fluorescence intensity corresponding to a single fluorophore. While possible, for example, through quantitative immunoblots,^128,142^ such calibration is complex and needs to be done specifically for each fluorophore, microscopy setting, and biological condition. However, even in the absence of calibration to obtain absolute amounts, it can be beneficial to convert raw pixel intensities to concentration or amounts, albeit using arbitrary units. This way, relative changes between cells and conditions can be interpreted more easily.

### B. Confocal microscopy

The depth of a typical confocal imaging plane is small compared to the height of most (eukaryotic) cells. Therefore, the local pixel intensity obtained from single confocal images closely corresponds to the local average fluorophore concentration in the confocal volume. To estimate cellular concentration, it is, therefore, necessary to average pixel intensity over representative z-stacks equally covering all parts of the cell. Overall, the fluorophore amount can then be obtained by multiplying average concentration with cell volume, ideally also estimated from 3D imaging.

### C. Epifluorescence microscopy

In contrast to confocal microscopy, signals obtained with epifluorescence microscopy also include significant contributions of fluorophores above and below the focal plane. Thus, depending on the objective used and the height of the cell imaged, the pixel intensity may correspond more closely to the amount of fluorophore at a given xy position (but summed over all z-planes) rather than local concentrations. Specifically, single epifluorescence images of budding yeast, focused on the center of the cell and obtained with standard microscope settings, collect most of the fluorescence emitted, which allows estimation of the total fluorophore amount as the summed intensity in the cell area.^8,143^ The experimental error associated with out-of-focus light outside the cell segmentation boundaries can further be reduced by using objectives with lower NA and/or magnification,^144^ which typically have a bigger depth of field. From the estimate of fluorophore amount, an average cellular concentration can then be obtained by dividing by the cell volume.

### D. Subcellular localization and co-localization

In addition to quantification of average cellular concentrations, fluorescence microscopy is often used to study subcellular localization. Fluorescent protein markers can also be used to highlight a specific organelle (e.g., histones for the nucleus, transmembrane proteins for the cellular periphery, septins and myosin for the bud neck, etc.). While the best image analysis strategy will depend on the exact question, many aspects discussed above will still be relevant for the quantification of subcellular signal: for example, local autofluorescence can vary depending on cellular compartment and biological context and the spatial dimension of the signal localization has to be considered to determine how the pixel intensity is linked to local concentration.

Finally, apparent co-localization of fluorescence signals is often used to infer (direct or indirect) physical interactions. While we refer to dedicated reviews for the details of co-localization analysis,^145^ we want to emphasize the importance of appropriate (negative) controls, and rigorous analysis and interpretation.

• Interpretable quantifications such as “concentration” or “amount” are preferable.
• Relationship with measured pixel intensity depends on microscopy setup, cell size, and cell geometry.

## VII. SINGLE CELL ANALYSIS

### A. Temporal alignment and data normalization

Once fluorescent data have been corrected for background and quantified as described above, the actual analysis and data interpretation can begin. Typically, a fluorescence live-cell imaging experiment yields time-resolved intensity data along with other measurements such as cell volume for dozens to hundreds of individual cells. From these single cell data, the experimentalist often wants to derive “typical” or “average” cellular behavior.^146,147^ To determine an average behavior, some sort of average has to be calculated from the individual single cell traces. However, for experiments involving asynchronous cell populations, this is complicated by the fact that the single cell traces start and end at different time points during the experiment. In addition, biological processes, such as the cell cycle, are typically heterogeneous in duration, resulting in cell cycle traces of different temporal lengths. Thus, to calculate an average, some sort of alignment may be needed. In principle, two different approaches can be used: (i) Single cell traces can be aligned at one characteristic time point, for example, the time of an external perturbation or the beginning of a cell cycle phase, which can be determined from the time-lapse data.^148–150^ This approach maintains time information and is, therefore, useful to study dynamics. However, due to the cellular heterogeneity, “synchrony” is lost gradually with the temporal distance to the alignment point, which can lead to averaging artifacts. (ii) Alternatively, a start and end point can be determined for each trace, and time can be scaled to allow averaging of multiple traces with different duration from start to end point.^151–153^ Obviously, the scaling of time will distort the dynamic information. However, such an approach might be a useful tool to extract common features of how the signal changes during a biological process.

**FIG. 4.**
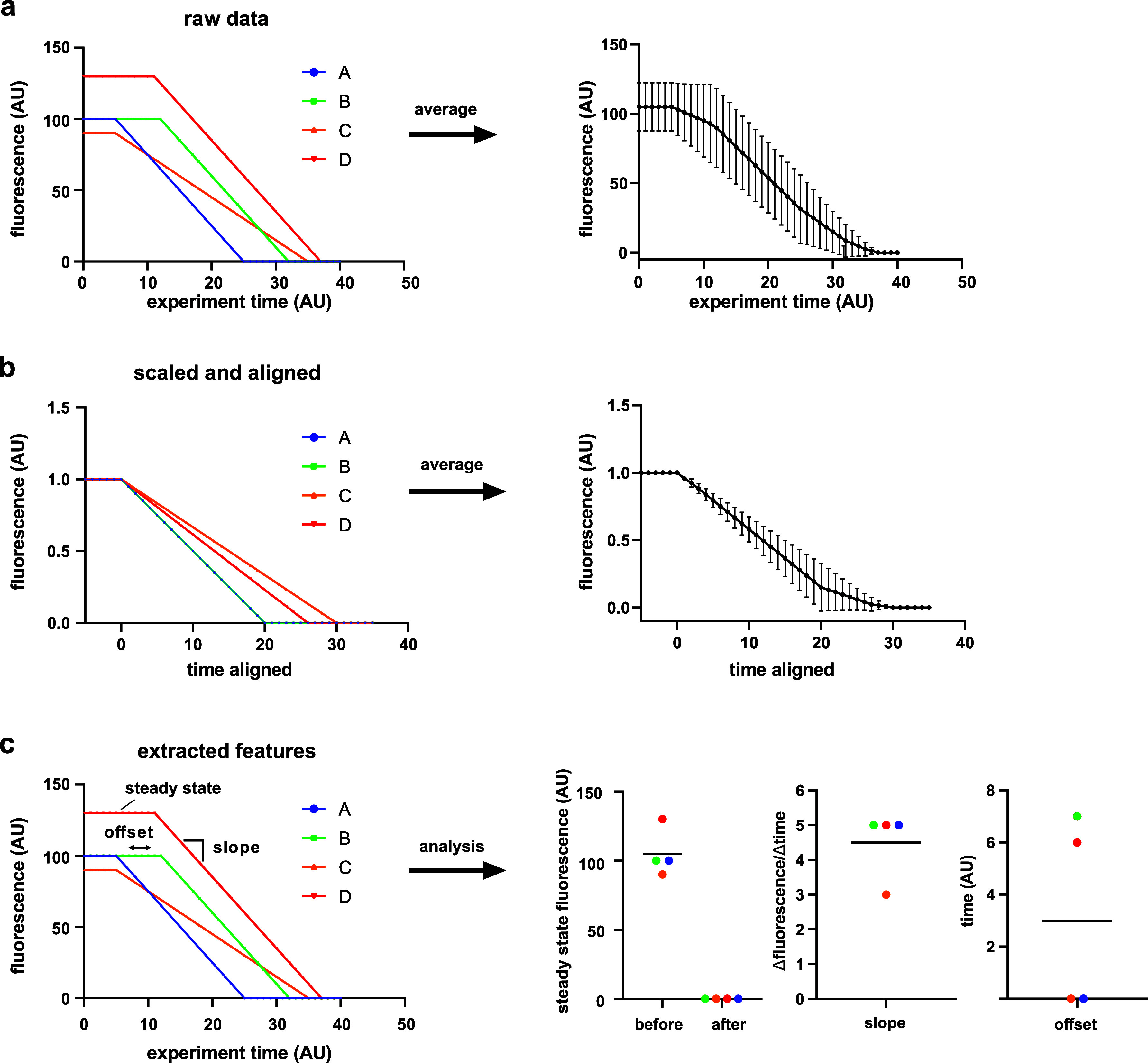
The effect of scaling and aligning. This toy example illustrates fluorescent signal from four cells (four colors) that experience a perturbation at t = 5. All cells respond by a linear decrease in the fluorescent signal. However, the cells respond with different delays and different slopes of the decrease. (a) If the raw data are averaged, the linearity of the response is lost. (b) The cells were aligned by their time of response and scaled by the initial steady state value. When these traces are averaged, the linearity is captured for the initial part of the curve but is then lost. The information on the distribution (error bars) is distorted. (c) Often it makes sense to extract features such as the slope directly from the single-cell data and then report on the distribution of these features.

Not in all cases, a meaningful average can be found. Instead, the relevant parameters of interest may need to be extracted on a single cell level, and distributions rather than simple means may then have to be interpreted to describe the biological phenomenon.^154^ To illustrate this, consider a scenario where the fluorescent signal linearly decreases from time point A to time point B, but the duration between A and B varies between cells. Since the slope of the decrease will be different in each cell, the average of the traces aligned at A will hide the fact that the decrease is linear. In contrast, scaling the duration between A and B will accurately describe the linearity, but the actual dynamic information is lost. Only calculating the slopes for each cell individually will reveal the distribution of the underlying dynamics (Fig. 4).

Similar to the need for temporal alignment and scaling, the normalization of the signal intensity may be necessary to extract the information of interest. For example, if the signal intensity varies dramatically between cells and the relevant information is a relative change in the signal, normalization—for example, on the mean value of each cell—may be useful to calculate fold-changes. In contrast, if the absolute strength of the signal matter, normalization will lead to a loss of important information.

In summary, temporal alignment and data normalization are useful and often necessary tools to extract interpretable information from single cell data. However, they always go along with a loss of information and a strong potential for artifacts. Thus, careful consideration should be given to how a specific averaging strategy translates to the underlying biology, and which strategy is, therefore, best suited.

### B. Data interpretation

Like any biological experiment, live-cell imaging experiments need to be carefully designed to ensure that the generated data can lead to biologically meaningful and statistically sound quantitative results. However, there are several aspects that are particularly relevant for quantitative live-cell imaging.^155,156^ First, it is important to clarify what can be considered a replicate, i.e., what defines the “n” of a statistical analysis of the data.^157^ One can easily generate a large number of observations (n) by analyzing many cells from one experiment. This high number of observations can lead to false confidence (“low p-values”) when comparing two different treatments or two different mutants that were not imaged within one setup (explained very nicely in detail in Ref. 158). The problem with this analysis is that single cells from one experiment are not necessarily independent and uncorrelated. For example, if there was a drift in the temperature of the incubator, all cells in one experiment may collectively grow more slowly than genetically identical cells grow a day later. Thus, it is important to perform multiple independent replicates and to understand and control for the sources of variability in single cell live-cell imaging data.

Variability and “noise” in live-cell imaging can be generated through at least three conceptually different sources: 1—true biological heterogeneity^159^ or cofounding biological parameters such as replicative age^160^), 2—purely technical noise in the signal generated by the hardware or during image quantification, and 3—technical noise that leads to biological responses. Biological and technical noise can be hard to distinguish, especially cases 1 and 3. For example, consider two colonies next to each other that exhibit different growth rates. This could be due to a complex inheritance phenomenon, or simply due to uneven flow in the cultivation device. Once an interesting observation is made, the key point is to carefully phrase alternative hypotheses^155^ and then to think carefully about how to setup appropriate controls, how to define and report replicates, and which statistical tests are appropriate for the data at hand.

Another potential pitfall of live-cell imaging is that only a small fraction of the population is sampled; during the multiple steps needed to setup the experiment, almost inevitably a subset of the whole population at several steps of the experiment is “chosen.” This can lead to biases, which significantly impact the conclusions drawn from the experiment.^155^ Biases can be already generated through the hardware, for example, if only cells of a given size are retained in the cultivation device. In the next step, the experimentalist typically chooses which fields of view to image over time. Finally, data analysis usually involves at least some manual intervention when choosing which cells to analyze. Additionally, complex phenotypes are often manually scored. Biases inherent to manual phenotype scoring can be circumvented by machine learning approaches. However, in this case, biases can come from the defined parameters or from the training sets. In summary, it is important to identify possible sources of biases, and plan controls accordingly. For a more comprehensive overview of possible biases and how to control for them, we refer the reader to two very good dedicated reviews.^155,156^

• Aligning or normalizing can be useful to determine typical behavior of cells but can discard information and generate artifacts.
• Extracting features from individual cell traces and analyzing their distributions can capture more information than averaging.
• Single cells from one experiment are not necessarily independent “replicates;” therefore, multiple experiments should be performed and reported transparently.
• Biological heterogeneity and technical noise are not always easy to distinguish, so careful controls probing the hypothesis are needed.
• Sampling biases can be introduced on multiple levels, including the available hardware, the choice of fields of view, the choice of which cells to analyze, and how to score phenotypes.

## VIII. REPORTING AND PRESERVING IMAGING DATA

To preserve (published) microscopy datasets, they should be findable, accessible, interoperable, and reusable (FAIR).^161^ For images to be findable and accessible, they need to be stored in a publicly accessible place under a permanent address.^162^ For images to be interoperable and reusable, they need to be linked to the biological experiment and to the metadata of the imaging setup. Furthermore, to recapitulate the key findings of a publication, the data processing pipelines should ideally be available.

### A. Data reporting

An essential part of reporting and publishing is to ensure reproducibility by describing an experiment as precisely as possible. For classical bench work, reporting protocols with precise descriptions of, e.g., chemicals, centrifugation steps or genetic strain engineering, is well established. By contrast, in many publications, the details provided about the microscopy setup and imaging conditions are often sparse, in part because commonly accepted reporting standards for quantitative imaging are still lacking.^163–165^ Nevertheless, the minimal imaging metadata required to reproduce the experiments for any quantitative analysis should contain the physical details about the filters used for each channel, the light source, the objective, microscope, and detector.^166^

An image channel is defined by a specific combination of filters and light source and is often just reported by a generic image name such as “GFP.” However, this information is not sufficient, and instead the minimal set of information for each channel should include: 1—the wavelength (ranges) for the excitation, beam splitter, and emission filters, 2—the center wavelength as well as the power of the excitation light (transmission and fluorescence), 3—the exposure time and imaging frequency, 4—the objective used with magnification, numerical aperture (NA), and immersion type and fluid, 5—the detector model with the pixel size, dynamic range (bit depth, i.e., 16 bits), binning (i.e., 1 × 1), and gain (for the CCD type of sensors) as well as the temperature and the type of sensor cooling, and 6—the microscope setup itself.^167^ An online check list tool can be found in Ref. 167. In addition to detailed reporting in the Methods section of any publication, this information should be stored in the raw image container (i.e., tiff) either directly or as additional (machine-readable) file. This will keep this crucial information connected to in the dataset,^168^ which is helpful especially when uploading to a repository as described below.

### B. Sharing raw images

Raw data are key to any (re-)analysis of an experiment and should be shared along with a publication.^169,170^ Therefore, more and more funding agencies and publishers require filling a data management plan (DMP) or data availability statement to ensure raw data access and preservation after publication.^171^ While in many “big data”—fields, such as genomics and proteomics, uploading data to public repositories is standard practice, this has been lagging behind in the imaging field. Storing and sharing imaging data it is not trivial^172^ because typical multi-channel, multi-position, time-lapse microscopy experiments often consist of ten to hundred thousand images and can come in different file formats.^173^ To handle these large datasets, dedicated online repositories are needed. Several such repositories are now established (Table II).^174^ Importantly, the deposited imaging data are only useful, if it is attached to precise metadata, detailing not only the biological experiment but also the technical setup as described above.

**TABLE II.** List of repositories.

Name	Data type (code, images, figures)	DOI	Storage limit	Description	Reference
Image data resource	Images	YES	Up to 1000 GB; above requires planning	IDR is a public repository of reference image datasets from published scientific studies. IDR enables access, search and analysis of these highly annotated datasets.	https://idr.openmicroscopy.org ^187^
Bio image archive	Images, text	YES	10 GB+ limit: n.a.	BIA stores and distributes biological images that are useful to life sciences researchers.	https://www.ebi.ac.uk/biostudies/BioImages/studies ^191^
Systems science of biological dynamics repository	Images, various file types	Yes	n.a. submit via email contact	SSBD is an open data archive that stores and publishes bioimaging and biological quantitative datasets that are associated with published or to be published studies.	https://ssbd.riken.jp/repository/ ^192^
The cell image library	Images, various file types	Yes	n.a. submit via email contact	CIL accumulates images of all cell types from all organisms, including intracellular structures and movies or animations demonstrating functions.	http://www.cellimagelibrary.org/pages/contribute
Figshare	Data of various file types	Yes	Up to 5 GB	Figshare a home for papers, FAIR data and nontraditional research outputs that is easy to use and ready now.	https://figshare.com
Zenodo	Data of various file types	Yes	50 GB, above requires contact	Zenodo is built and developed by researchers, to ensure that everyone can join in Open Science and allows to share data.	https://zenodo.org
Bio image	AI models, applications, datasets	Yes	See Zenodoo	Bio image—A collaborative effort to bring AI models to the bioimaging community. Data is hosted on Zenodoo.	https://bioimage.io

Note: An up-to-date list of research data repositories beyond this table can be found here: https://www.re3data.org/.

### C. Sharing code and image processing pipelines

To be able to reproduce an image-based analysis, not only the raw (image) data should be accessible but also the code used for the analysis.^175^ Often an analysis consists of several steps using different software tools to process the raw images such as deconvolution, segmentation, tracking, and quantification.^176^ These steps can be done manually or in an automated way using scripts.^177,178^

Manual interactions by clicking through different software tools are sometimes useful but are hard to document and reproduce by others. Therefore, open-source tools that can be interfaced by scripts^116,179,180^ or integrated in larger pipelines are preferable.^97,178^ Ideally, these scripts should include all steps or instructions necessary to extract the information from the images to reproduce figures and tables.^181,182^ To facilitate sharing and re-use of scripts, it is good practice to follow established coding and style guidelines of the programming language used and to provide instructions on what needs to be run in which order.

### D. Toward interactive data—Enabling fast validation and re-use

While publishing data and associated analysis openly according to the FAIR principles should become the standard, a key question is still how to promote the re-use of published data for novel biological questions.^183^ Even if the data are well documented and organized, it may not be reused due to the fact that the entry level is often still too high (for example, because the analysis requires an expensive license, special hardware, or is written in an unfamiliar programming language). One solution to lower the entry level is to make the data or its analysis, respectively, interactive. This can be achieved with computational notebooks, which allow to execute code snippets and visualize its result in one document instantaneously^184^ or using workflows.^178^ Computational notebooks are available for most popular scripting or programming languages.^185^ One advantage is that they can directly be executed from public code repositories, such as GitHub, through services, such as Binder,^186^ without the need for dedicated hardware. This allows anyone to freely run and interact with a certain analysis directly online. However, while this typically works well for code and small datasets, working with large imaging datasets is still a challenge in terms of bandwidth and due to the fact that the data are often hosted in different repositories. Promising work is ongoing to address this remaining challenge^187^ to link (meta-) information from electronic laboratory notebooks and laboratory information systems (ELN-LIMS) and data with code repositories to make complex analysis pipelines truly accessible and interactive from anywhere.

• Ensure reproducibility by reporting details on the optical filters, the light source, the objective, microscope, and detector. Report physical parameters using SI units rather than just brand names.
• Attach precise metadata of the microscopy setup to your image files. Most modern image acquisition software allows entering these details prior to an experiment.
• Publish data FAIR. Use open well-established data formats, and wherever possible, open accessible analysis software.
• Try to separate code from data repositories and make them available as an interactive workflow wherever possible.

## IX. CONCLUSION

Since the discovery of fluorescent proteins, live-cell imaging has become a powerful and popular technology driving research in many live science fields ranging from molecular and cell biology to systems biology and biophysics. A successful live-cell imaging experiment requires many steps from setting up the hardware to acquiring images, data analysis, and reporting. Each of these steps comes with challenges and potential pitfalls. Still, consensus best-practice procedures for each of these steps do not exist to date. In fact, general consensus may even be hard to find due to the many different applications and biological questions at hand. Rather than providing direct guidelines on best practices, we, therefore, aimed in this review to make the readers aware of the many parameters that need consideration when setting up an imaging pipeline. Most importantly, whatever tools and parameters are chosen—starting with the hardware setup all the way through data analysis, they need to be reported transparently. Only then can live-cell imaging experiments be recapitulated and reproduced by others, all the way from idea to image to insight.

While some of the recommendations we made here are relatively straightforward to implement, others—especially with regard to data sharing and accessibility—will require a significant effort by the community to establish as standard. In light of this, we readily admit to not having always lived up to our own standards.

## X. METHODS

In Fig. 3, a prototrophic budding yeast strain expressing Msn2-GFP (haploid W303 strain, mating type a, MSN2-yEGFP-HIS3, WHI5-mCHERRY-KANMX, ADE, TRP, LEU, URA) was grown in two lanes (L1 and L2) of a microfluidic plate (CellAsic Onix2, Merck) at 30 °C (Cage incubator H201, Oko-lab) with constant flow of synthetic minimal medium (Smin) + 1% glucose. Smin consists of 1, 7 g/l YNB powder (U.S. Biological), 5 g/l ammonium sulfate (Merck), and 1% d-glucose (Sigma-Aldrich) was added. From the onset of microfluidic flow, cells were imaged under an epifluorescence microscope (Ti-2, Nikon). The condenser turret TI2-C-TC-E was used with an LWD lens (Nikon, NA 0.52). Koehler illumination was calibrated before the experiment. 14 h of time-lapse images were taken every 5 min in 11 xy-positions for both L1 and L2. For each spatial and time position, phase contrast (PH3 ring, Nikon) and fluorescent (GFP, 475 nm excitation) images were taken. To induce photo-stress, L1 was imaged with an excess of blue light (390 nm, 500 ms, 20% intensity) and L2 with yellow light (575 nm, 1 s, 30% intensity). These intensities and exposure times of stress light were selected to induce similar levels of photo-stress between L1 and L2. In preliminary experiments, the relation between light quantities and cell death was studied. Cell death rates at the end of the experiment were used as a readout for levels of photo-stress.

The excitation illumination was based on LEDs (Lumencor Spectra X). For blue light stress, the quad-filter set 89 402 (Chroma, excitation 391–32/479–33/554–24/638–31) was used with the blue-violet LED (390 nm). For the yellow light, filter set 89 403 (Chroma, excitation 436–28/506–21/578–24/730–40) was used with the yellow LED (575 nm). For the GFP channel, the same filter set was used with the blue-cyan LED (475 nm). Using a 40× oil phase contrast objective (CFI PLANAPOCHROMAT DM 40× Oil NA 1.0, Nikon) with type F immersion oil (n = 1.518, Nikon), cells were imaged with 16 bits using a Photometrics Prime 95B CMOS camera (11 × 11 *μ*m pixel size; 13.2 × 13.2 mm sensor area; no binning (1 × 1); 50 000:1 dynamic range).

The microscope was controlled, and data acquired with the proprietary NIS-elements (Nikon) software. In NIS-Elements, the images were converted to single files in the TIFF format. In the same step, images were scaled to 8-bit. This enables subsequent analysis in matlab (The MathWorks, Inc.) based on an automated segmentation and fluorescence signal extraction tool.^93,188^ To define cell death, we first subtracted the extracellular medium background from all data. Then, cells were counted as dead when the signal of the whole cell (normalized by area) was more than twice as bright (arbitrary units) as the mean of ten unstressed dividing cells. In contrast, Msn2 signal peaks were defined by a ≥5-fold increase in the nuclear fluorescence signal. The nucleus was identified by applying a two-dimensional Gaussian fit around the brightest fluorescence pixels. Budding was used as an indicator for cell cycle entry.^154^

## Data Availability

The data and analysis code that support the finding of this study are available at doi://10.5281/zenodo.6406926.
